# Children’s Eating Habits, Physical Activity, Sleep, and Media Usage before and during COVID-19 Pandemic in Poland

**DOI:** 10.3390/nu13072447

**Published:** 2021-07-17

**Authors:** Edyta Łuszczki, Anna Bartosiewicz, Iwona Pezdan-Śliż, Maciej Kuchciak, Paweł Jagielski, Łukasz Oleksy, Artur Stolarczyk, Katarzyna Dereń

**Affiliations:** 1 Institute of Health Sciences, Medical College of Rzeszów University, 35-959 Rzeszów, Poland; abartosiewicz@ur.edu.pl (A.B.); kderen@ur.edu.pl (K.D.); 2 Institute of Physical Culture Sciences, Medical College of Rzeszów University, 35-959 Rzeszów, Poland; ipezdan@ur.edu.pl (I.P.-Ś.); mkuchciak@ur.edu.pl (M.K.); 3 Department of Nutrition and Drug Research, Institute of Public Health, Faculty of Health Sciences, Jagiellonian University Medical College, 31-066 Krakow, Poland; paweljan.jagielski@uj.edu.pl; 4 Orthopaedic and Rehabilitation Department, Medical University of Warsaw, 02-091 Warsaw, Poland; loleksy@oleksy-fizjoterapia.pl (Ł.O.); artur.stolarczyk@wum.edu.pl (A.S.)

**Keywords:** children and adolescents, COVID-19 pandemic, dietary patterns, media usage, physical activity, sleep

## Abstract

The COVID-19 pandemic has caused huge changes in people’s lifestyle, health, and social relationships. This situation has had an impact on children and adolescents, affecting their health, intellectual, physical, and emotional development. The survey aimed to compare eating behaviors, level of physical activity (PA), hours of sleep, and screen time among Polish children and adolescents aged 6–15 years before and during the COVID-19 pandemic. We obtained self-reported data from 1016 participants at two measurement points before and during the COVID-19 lockdown in Poland to examine the influence of the lockdown and the distance learning on PA, dietary habits, sleep, and media usage of children and adolescents aged 6–15 years. The study identified dietary differences and changes in daily activity patterns (reduced sleep duration with higher sleep quality and reduced physical activity). Additionally, the increase in general media usage was observed during the pandemic alongside a reduction in smartphone usage. Together, the findings indicate increased sleep, physical activity, and reduced media usage and screen time among Polish children and adolescents during the COVID-19 pandemic.

## 1. Introduction

The COVID-19 pandemic has caused huge changes in people’s lifestyle, health, and social relationships. The worldwide lockdown has affected socioeconomics, education, health care, and mental illness [1,2,3]. The situation has had a large influence on children and adolescents, impacting their health, mental, physical, and emotional development [4]. Many children and adolescents in the developed world were already sedentary before the pandemic, spending a lot of time using various technical devices. As reported by Lancet, 81% of students aged 11–17 were not physically active enough before the pandemic [5]. However, the demands of everyday life were a mobilization to leave home, go to school, meet with friends, and participate in various activities outside the classroom. Due to the pandemic situation and the need to avoid infection, the previous activities were completely reduced, and home quarantine was conducive to a significant increase in computer time. Children and young people faced with the pandemic experienced such enormous changes in their lives as never before. The COVID-19 pandemic has led to rapid, unprecedented changes in the lives of billions of children and adolescents around the world [6,7]. Almost 90% of children and adolescents, approximated as 1.5 billion youth worldwide, have been obliged to stay at home. Apart from studying at school, children undertake many additional activities, such as horse riding, swimming, dancing, learning foreign languages, etc. The limitation of these activities also limited contact with friends, which significantly increased efforts to maintain relationships through social media and various types of communicators [8]. It significantly influenced their health, including nutrition, physical exercise, sleep, and social functioning, increasing their media use [9].

Governments in Europe responded with various measures aimed to slow down the transmission of COVID-19. Those restrictions, which included the closure of schools, playgrounds, and parks, significantly reduced chances for children and adolescents to maintain a healthy lifestyle with physical activity (PA). Children lost opportunities for physical activity in school and outside of school. Recommendations for children and adolescents indicate that they should accumulate >60 min of moderate to vigorous-intensity physical activity and involve <2 h of screen time/day [10]. Results from countries such as Canada, the U.S., and China demonstrated a reduction in physical activity and rise in screen time during the first wave of the COVID-19 pandemic [11,12,13,14]. In another study, only <10% of children 5–17 years have achieved physical activity recommendations [15]. Nevertheless, there is a lack of knowledge about the impact in Europe, including Poland.

The first confirmed case in Poland was diagnosed on 4 March 2020. An epidemic emergency state was professed on 20 March pursuant to a Regulation of the Minister of Health [16]. Control measures were implemented on 10–12 March, cancelling mass events and closing kindergartens, schools, universities, and offices; then, they were strengthened on 25 March, limiting religious gatherings and forbidding non-essential travel.

The literature suggests that social restrictions required to lessen the spread of COVID-19 have increased inactive behaviors [17], deteriorated sleep patterns [18], and decreased physical activity [19]. Children and adolescents had to make a lot of changes in their everyday lives, including home confinement, school closures, and social distancing rules [20]. The closure of sport clubs as well lower physical activity and staying at home for long hours may result in body mass increase in children and obesity development. However, in developing countries such as Poland, the opposite may appear: the economic aspect of COVID-19 may decrease parents’ abilities to provide food supplies for their children and can lead to malnutrition [21]. Additionally, they do not receive meals at school during the lockdown, which can increase the occurrence of malnutrition [22]. Therefore, the hypothesis was that the COVID-19 pandemic caused undernutrition in low and low–medium income countries, especially in children [23].

However, results from the online survey including 1097 people in Poland showed that 43% of people ate more, while 52% ate more snacks during the nationwide quarantine. Almost 30% of adults reported weight gain [24].

Sedentary behavior is a risk factor for cardiometabolic disease also in adults and children [25]. Guidelines recommend ≤2 h of recreational screen time/day for 5–17-year-olds [26]. However, almost 50% of children in the U.S. aged 6–11 spent >2 h/per day on sedentary behavior such as playing video games, watching TV, and using smartphones before the pandemic [27]. Similar results were observed in Poland [28]. Data from studies conducted during the COVID-19 pandemic presented significant (20–66%) increases in media usage [29].

Sleep disturbances may have an impact on health, attention span, and immune function [30]. For children 6–15 years old, 9–11 h of uninterrupted sleep are recommended [26]. Moore et al. showed those children were sleeping more hours compared to the period before the COVID-19 pandemic [12]. Likewise, Pietrobelli et al. reported that hours of sleep have risen by 0.65 h per day during the pandemic compared to the year 2019 in Italian children [13]. However, sleep quality has not been studied within the context of the COVID-19 pandemic among children and adolescents.

To understand the effects of the COVID-19 pandemic on children’s and adolescent’s nutrition, physical activity, sleep, and media use, we administered an online survey one year after our previous study conducted before the pandemic. The survey aimed to compare eating behaviors, level of physical activity, hours of sleep, and screen time among Polish children and adolescents aged 6–15 years before and during the COVID-19 pandemic.

## 2. Materials and Methods

### 2.1. Study Design and Sample

This is a cross-sectional study that was conducted among 1016 participants using a survey technique. The self-reported data were carried out at two measurement points before (pre) and during (peri) the COVID-19 lockdown in Poland to examine the effect of the lockdown and the distance learning on PA, dietary habits, sleep, and media usage of children and adolescents aged 6–15 years.

This study consisted of two parts (pre and peri). The results of the first study have been published [31]. The part before the pandemic was conducted at the turn of February and March 2020 in the randomly selected schools in the Podkarpackie Voivodeship in Poland. The following inclusion criteria for the study were used: aged 6–15 years, the attendance of one of the selected schools, and parents’ acceptance of their children’s participation in the study. Currently, Poland has an eight-year education system in primary schools for children aged from 6 to 15 years.

The pre-study group consisted of 376 pupils (187 boys and 189 girls) aged 6 to 15 years and was a representative sample of the population of southeast Poland. The study methodology has been published in detail [31].

Assuming a 5% error threshold and a test power of 0.95 for this population size, the number of children included in the pre-pandemic study was sufficient. In the second measurements point (peri) carried out during the pandemic, by increasing the size of the group to 641 participants, it was possible to reduce the error threshold to 4%, i.e., the test power was 0.96. The number of children participating in the study is representative of the population of children from the southeastern region of Poland.

One year later, from 1st February until 2nd March 2021 during the COVID-19 pandemic and lockdown in Poland, the second part of the study was conducted. The same methodology with the same questionnaire was used. Target recruitment was a minimum of 376 children collected via convenience sampling in the same part of southeast Poland (Podkarpackie Voivodeship). Children in the defined age group who resided in the region were eligible to participate. After obtaining the consent of the directors of all primary schools and the mayor of the randomly selected city for conducting the research, we sent the information to the children and their parents/guardians via the electronic journal. The message contained information about the planned research and a consent form for parents/guardians for their child to participate in the study. The participants were assured of their voluntary participation and of the anonymity of the study. The total number of the study group during the pandemic was 641.

### 2.2. Weight, Height, and BMI

In the pre-study, the height was measured three times with an accuracy of 0.1 cm by means of a Seca 213 portable stadiometer, and the weight was measured assessed with an accuracy of 0.1 kg using a body composition analyzer (BC-420, Tanita, Tokyo, Japan) [31]. In the peri study, weight and height were declared by the person who filled in the questionnaire during the COVID-19 pandemic.

BMI was calculated as a ratio of weight to height (kg/m^2^). Centile grids for BMI according to age, sex, and height developed under the project “Developing standards of blood pressure in children and adolescents in Poland, OLAF” were used in our study to define childhood underweight, normal weight, overweight, and obesity [32].

### 2.3. Questionnaire Data

The questionnaires for younger children (6–12 years old) were completed by their parents/guardians, while the adolescents (13–15 years) filled in the online questionnaire on their own. A personal survey link was sent to each parent or/and child. The responses of the study participants were automatically stored on the hard drive of the company computer and on a mass storage media. The questionnaires received were checked at the survey center for completeness, and missing questions were added by conducting an interview. There were two additional interviews with participants, which lasted about 15 min. The questionnaire used in the study was divided into four sections and covered the information referring to the children’s lifestyle, eating and sleeping habits, the use of technical devices and the Internet, and socio-demographic data. The time needed to complete the questionnaire was approximately 25 min. An example of the questionnaire is attached as a supplementary file. After completing the research, all the data were deposited in the Rzeszow University repository.

To assess the nutrition habits of the respondents, the modified Food Frequency Questionnaire (FFQ-6) was used [33]. Next, questions were connected to the child’s sleeping behavior: the amount of time spent sleeping at night and during the day and sleep quality. There were questions: What is the amount of time you sleep during a 24 h period on school days? What is the amount of time you sleep during a 24 h period on weekends? During the past month, how would you rate your sleep quality overall? (Possible answers were: Very good, Fairly good, Fairly bad, Very bad). The third part of questionnaire was related to the use of technical devices and the Internet by children [34]. Physical activity was assessed with the question: Over the last week, how many days have you performed 60 min or more of physical activity that increased your breathing rate? This may include doing sport (football, volleyball, basketball etc.), exercises, brisk walking, or cycling for recreational purposes or on the way to these places. The possible answers were from 0 to 7 days. Details about the questionnaire were described in a previously published study [31].

### 2.4. Statistical Analysis

Results of the study were developed using descriptive statistics: number (N), percentage (%), mean (𝑥̅), median (Me), and standard deviation (SD). The Shapiro–Wilk test was performed to check the normality of the data. A non-parametric Mann–Whitney U test was used. The analysis of variables having the character of qualitative data was carried out with the Pearson chi-square test. The statistical analyses were performed using PS MAGO PRO 6.0 (IBM SPSS STATISTICS 26). Statistical significance was set at *p* < 0.05.3. Results

## 3. Results

### Characteristics of the Study Group, Physical Activity, and Sleep

The study was conducted among 1,016 children at two measurement points before (pre, *n =* 376) and during (peri, *n =* 640) the COVID-19 pandemic in Poland, including 495 boys (48.7%) and 521 girls (51.3%). It was found that children in the pre-group were more physically active than children in the peri group (*p <* 0.0001). The average value (Me) of the level of physical activity before the pandemic was 4 days with 60 min or longer physical activity, while during the pandemic, it dropped to 3 days. In addition, a recommendation for physical activity for children was met by 12.3% of children before the pandemic and 9.2% during the pandemic, but this difference was not significant (*p* = 0.1220).

Significant differences in the duration spent sleeping during a 24 h period were observed between the two groups (*p* = 0.0009). Both on school days and on weekends, a decrease in sleep time was noticed. Sleep time on weekdays dropped from 8.83 to 8.55 h and on the weekend from 10.11 to 9.52 h. However, we found that the sleep quality was significantly higher during the pandemic (*p* = 0.0323).

The differences between the pre and peri groups are shown below (Table 1).

**Figure 1:**
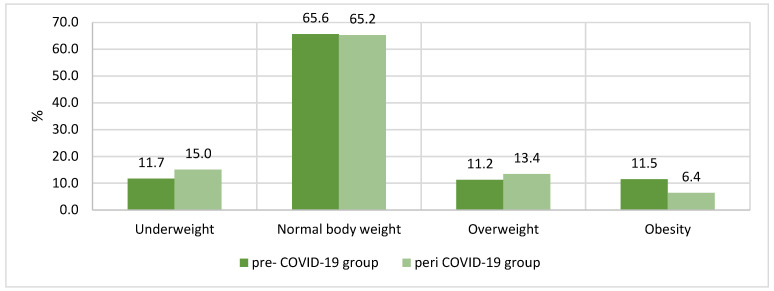
Body weight categories based on BMI centiles in the groups pre- and peri COVID-19 (*p* = 0.065).

Figure 1 presents the body weight categories based on BMI centiles. There were no significant differences between the groups pre and peri COVID-19.

Characteristics of media usage in the groups pre- and peri COVID-19 are presented in Table 2. Significant differences in time of watching movies or programs on the Internet or TV were found between children from the group before the pandemic and during the pandemic. During the pandemic, children often watched significantly more movies or programs on the Internet or TV on weekdays but not on the weekend. On the weekdays, the percentage of children who watched more than 6 h per day increased from 1.3% to 5.1% (*p* = 0.0319). Moreover, children during the pandemic significantly more often spent time on the weekdays on playing games (1.29 h vs. 1.64 h; *p* < 0.0001). However, using a smartphone on a regular day has decreased during the pandemic from 3.14 to 2.81 per day (*p* = 0.0016).

In Table 3, the difference between the two groups in frequency of food consumption is shown. It was found that the frequency of consumption of most products has decreased ($\bar{x}$; pre vs. peri): legumes (1.63 vs. 1.36), potatoes (2.82 vs. 2.51), fruit juices (3.28 vs. 2.77), carbonated sugar sweetened drinks (1.77 vs. 1.40), diet carbonated drinks (1.43 vs. 1.22), cold cuts and preserved, ready-to-cook meat product (2.94 vs. 2.61), fast food (1.76 vs. 1.39), nuts and seeds (2.00 vs. 1.76), and snacks (1.95 vs. 1.56).

However, especially in milk products consumption, an increase has been observed (*p* < 0.05). Additionally, the consumption of boiled, grilled, oven baked, raw fish, poultry, and meat also increased (*p* < 0.05). It was also noticed that the consumption of sweets increased (*p* = 0.0371).

## 4. Discussion

Our results present that there were differences in eating behaviors, level of physical activity, hours of sleep, and screen time among Polish children and adolescents aged 6–15 years before and during the COVID-19 pandemic. To our knowledge, this is the first study conducted in Poland among children before and during the COVID-19 pandemic and assessing the change in media usage, sleep time, eating habits, and physical activity.

Our results show a decline in PA among children aged 6–15 years during the pandemic in contrast to the time before the lockdown. The average number of days with 60 min or longer physical activity dropped from 3.89 to 3.30. Data from the literature investigating changes in children’s PA are limited. The first preliminary results indicate a downward trend in PA levels [12,19]. The main reason is probably social restriction such as remote learning. In addition, parental limitations due to ‘shelter-at-home’ recommendations and working from home may create difficulties in keeping children physically active. In a study of 1472 Canadian youth, only 3.6% of children aged 5–11 and 2.6% aged 12–17 years met the recommendation of achieving 60 min of moderate-vigorous physical activity/per day during the COVID-19 pandemic [12]. In our study, this recommendation was met by 9.2% of children (in contrast to the 12.3% before pandemic). In a study of 97 South Korean parents, 94% described a reduction in daily physical activity of their children during the COVID-19 pandemic [29].

Our study demonstrated the change in the sleep duration between the period before and during COVID-19 pandemic. Lack of sleep may have an impact on emotional health, attention span, and immune function [18,30]. Insufficient sleep can increase the risk of cardiometabolic disease in both children and adolescents and results in mood swings anxiety, which may be intensified by poor mental health during the COVID-19 pandemic [30,35]. The Australian Department of Health recommends between 9 and 11 h of sleep for children aged 5–13 years and 8–10 h of sleep for adolescents aged 14–17 years [36]. Our results show the decrease in the sleep duration both on the weekday and on the weekend. The average duration spent sleeping (including naps) was 8.83 h before and 8.55 h during the pandemic on the schooldays. On the weekend, the average duration spent sleeping also decreased from 10.11 to 9.52 h. However, the sleep quality slightly increased. The decrease of the sleep duration can be explained by unscheduled sleep during the COVID-19 pandemic, usually without parents’ control. Children spent more time in front of the computer/tablet/television and extensive use of electronic devices was noticed; therefore, it could cause a delay in bedtime and as a consequence shortened sleep duration. In Poland, the distance learning was according to the school lessons plan, and the lessons were usually held from 8.00 a.m. Moore et al. showed that children were sleeping more hours than they had been prior to the COVID-19 pandemic [12]. Likewise, Pietrobelli et al. noticed that sleep increased by 0.65 h per day during the pandemic among Italian children compared to the period before the pandemic [13]. This difference was very small, but changes between our results and other studies can be explained by the specific sleeping and waking up routines among countries. In Poland, parents usually get up very early to work (both remotely and traditionally), which is why they often wake their children. However, data on sleep duration are inconsistent across the literature. Israelian research on infants and children presented inconsistent changes in sleep duration, both an increase and decrease or no changes [37].

Digital media and devices are an integral part of the modern world. During the pandemic, the use of these devices has become a condition of everyday functioning not only of adults but also of children [38]. Despite the potential benefits, excessive and inappropriate use of technology may have an effect on children’s development and health. According to the previous research, there is a link between increased screen time and a greater risk of health complications, mental health issues, and the negative effects of cognitive, linguistic, social, and emotional development [39,40,41]. Our results showed significant differences in the time spent by children using the Internet and TV between groups before and during the pandemic. Moreover, the increase in time in front of the monitor mainly concerned days during the week, not on the weekend, as it was before the pandemic. The percentage of children who spent more than 6 h per day watching TV and using the Internet increased from 1.3% to 5.1% (*p* = 0.0319). The amount of time spent on computer games also increased (1.29 h vs. 1.64 h; *p* < 0.0001), and this relates to weekdays. However, using a smartphone on a regular day has decreased during the pandemic from 3.14 to 2.81 per day (*p* = 0.0016). According to the American Academy of Pediatrics report from 2019, even before the pandemic, children spent a lot of time in front of the screen; children aged 8–12 spent an average of 4.5 h a day on screens, while teens aged 13–18 spent 6.5 h a day [42,43]. The pandemic and the resulting closures of schools, sports, and most extra-curricular activities only increased our dependence on digital tools, thereby increasing the children’s time spent in front of the monitor. As reported by the Statista report, one of the largest statistical websites in the world, in the USA, the amount of screen time spent by children of all age groups increased by an average of 4 h a day. The pandemic has increased the use of electronic devices among American children and teens, whose screen time is now twice as high as it used to be [44]. Similar results were obtained in a German study of 1717 participants at two measurement points before and during the pandemic, which was published in the journal *Nature* [45]. Schmidt et al. showed that watching TV and recreational Internet browsing were significant factors that influenced the extension of children’s time in front of the monitor [45]. Our results are also consistent with an experimental longitudinal study conducted among children and adolescents in China [14] and in Spain [45]. It shows that the special circumstances during COVID-19 have caused many changes in children’s media and electronic device use habits. It is extremely important to use the available evidence and prevent unhealthy screen time that may affect health and well-being among children.

Our study found that the restrictions as a result of the COVID-19 pandemic induced reductions in the consumption of certain foods such as legumes, potatoes, fruit juices, carbonated sugar, sweetened drinks, diet sodas, meats and canned food, ready-to-cook meat products, fast food, nuts and seeds, and snacks. There was an increase in the consumption of dairy products. Additionally, the consumption of cooked, grilled, baked in the oven, raw fish, poultry, and meat as well as sweets increased. The consumption of fresh vegetables and fruit was lower, but no statistically significant differences were observed. A Spanish study found significant reductions in fruit and vegetable consumption during the COVID-19 pandemic in a subset of children aged 3 to 5 years. In the group of children aged 6–16, it was also smaller, but no statistically significant differences were observed [46]. Similarly, a study with Italian children and adolescents whose schools closed due to the COVID-19 pandemic showed a higher consumption of red meat and potato chips [13]. General eating habits may have deteriorated during the pandemic. In addition, less food consumption outside the home during the pandemic has been associated with changes in dietary quality, including lower consumption of fast food. On the one hand, restrictions resulting from leaving the house only to perform permitted activities could have resulted in limited access to, for example, fresh fruit and vegetables. However, staying home and closing businesses may have reduced exposure to processed foods such as fast-food restaurants.

There were a number of potential limitations present in this study that should be considered when interpreting the results. Self-submitted and parental questionnaires were used to assess children’s eating habits, physical activity, sleep, and media use. Self-reported data are likely to recall bias and social desirability. Additionally, it was the cross-sectional study from two different measurement points. It would be a more helpful and meaningful study using the within-subjects method rather than between subjects. Repeated measurements (pre/peri COVID-19 groups) strengthened the study but did not allow for a population-specific generalization of the changes in that period. The limitation is also the lack of details about the intensity of PA.

## 5. Conclusions

In conclusion, our study comprehensively examined eating habits, physical activity, sleep, and media usage differences between children before the COVID-19 pandemic and one year later during the pandemic. The study identified dietary differences (e.g., reductions in the consumption of fruit juices, carbonated sugar, sweetened drinks, diet sodas, meats and canned food, fast food, snacks and increase in the consumption of dairy products, fish, poultry, meat, and sweets) and changes in daily activity patterns (reduced sleep duration with higher sleep quality and reduced physical activity). Additionally, an increase in general media usage was observed during the pandemic alongside a decrease in smartphone usage. The obtained results indicate increased sleep, physical activity, and reduced media usage and screen time among Polish children and adolescents during the COVID-19 pandemic.

## Figures and Tables

**Table 1:** The pre- and peri groups differences.

Variable			$\bar{x}$	SD	Me	Min	Max	*p*
Age (years)	pre		10.51	2.13	10.00	6.00	15.00	0.1032
	peri		10.79	2.02	10.00	6.00	15.00	
Height (cm)	pre		148.33	13.93	148.00	116.00	183.00	**0.0255**
	peri		146.48	13.93	146.00	110.00	197.00	
Weight (kg)	pre		42.40	14.38	39.80	19.00	107.30	0.0536
	peri		40.58	13.36	38.00	19.00	97.00	
BMI (kg/m^2^)	pre		18.78	3.83	17.90	12.10	39.90	0.2099
	peri		18.46	3.58	17.78	11.83	34.67	
Days with 60 min or longer physical activity	pre		3.89	1.89	4.00	0.00	7.00	**<0.0001**
	peri		3.30	2.07	3.00	0.00	7.00	
Duration spent sleepingduring a 24 h period (h)	pre	Weekdays	8.83	1.64	9.00	4.00	13.45	**0.0009**
	peri	Weekdays	8.55	1.17	9.00	3.00	12.00	
	pre	Weekend	10.11	1.45	10.00	5.00	15.30	**<0.0001**
	peri	Weekend	9.52	1.36	9.50	4.00	15.00	
Sleep quality	pre		1.70	0.68	2.00	1.00	4.00	**0.0323**
	peri		1.78	0.65	2.00	1.00	4.00	

$\bar{x}$—arithmetic mean, Me—median, SD—standard deviation, Min—sample minimum, Max—sample maximum, *p*—*p* value, indicate significant values (*p* < 0.05). Bold values denote statistical significance at the p < 0.05 level.

**Table 2:** The media usage characteristics in the groups pre- and peri COVID-19.

**Variable**			$\bar{x}$	**SD**	**NAA** **(%)**	**<30 min (%)**	**30 min–2 h (%)**	**≈** **2–3 h** **(%)**	**≈** **3–6 h** **(%)**	**>6 h (%)**	***p***
Time of watching movies or programs on the Internet (on an iPad, tablet, computer, smartphone) or TV per day [h]	pre	Weekdays	2.12	1.00	11.7	0.0	61.1	20.5	5.3	1.3	**0.0319**
	peri	Weekdays	2.34	1.12	4.4	14.0	44.0	23.4	9.0	5.1	
	pre	Weekend	2.81	1.04	4.3	0.0	34.4	37.6	18.9	4.8	**0.0276**
	peri	Weekend	2.70	1.10	2.3	8.0	35.9	31.5	16.2	6.1	
Time of playing games (on your computer/game console/smartphone/iPad etc.) per day [h]	pre	Weekdays	1.29	1.08	27.7	30.1	31.5	7.2	2.7	0.8	**<0.0001**
	peri	Weekdays	1.64	1.23	20.3	27.8	29.5	14.7	5.9	1.9	
	pre	Weekend	1.94	1.23	13.3	21.3	37.6	16.0	9.1	2.7	0.0599
	peri	Weekend	2.11	1.29	11.4	20.4	34.2	18.6	11.1	4.4	
**Variable**		$\bar{x}$	**SD**	**I Don’t Have Access (%)**	**<5** **Times a Day (%)**	**6–10 Times aDay (%)**	**11–20 Timesa Day (%)**	**21–50 Times** **a Day (%)**	**51–100 Timesa Day (%)**	**>100 Times** **Per Day (%)**	***p***
Using a smartphone on a regular day	pre	3.14	1.65	18.4	22.1	22.1	14.7	13.1	6.1	3.5	**0.0016**
	peri	2.81	1.58	23.6	26.8	20.0	13.3	9.5	4.4	2.5	

NAA—not at all, $\bar{x}$—arithmetic mean, SD—standard deviation, *p*—*p* value, indicate significant values (*p* < 0.05). Bold values denote statistical significance at the p < 0.05 level.

**Table 3:** Differences in frequency of food intake between children before and during the pandemic.

Selected Food Items		$\bar{x}$	SD	Never/Less than Once a Week (%)	1–3 Times a Week (%)	4–6 Times a Week (%)	1 Time Per Day (%)	2 Times Per Day (%)	3 Times Per Day (%)	4 or More Times Per Day (%)	*p*
Legumes (e.g., beans, lentils, chickpeas)	pre	1.63	1.14	60.5	29.6	4.5	1.9	0.8	0.3	2.4	**0.0009**
	peri	1.36	0.65	68.8	28.7	1.6	0.3	0.2	0.2	0.3	
Potatoes (cooked)	pre	2.82	1.27	4.0	44.8	34.7	8.5	2.1	1.1	4.8	**<0.0001**
	peri	2.51	1.01	5.8	55.1	29.3	6.4	0.6	0.9	1.9	
Raw vegetables (mixed salad, carrot, fennel, cucumber, lettuce, tomato)	pre	2.90	1.57	16.8	29.3	30.1	9.6	4.8	4.0	5.3	0.4869
	peri	2.85	1.29	12.5	35.6	19.5	24.6	4.2	2.2	1.4	
Fresh fruits	pre	3.55	1.77	8.8	24.0	25.9	12.5	12.3	6.1	10.4	0.7831
	peri	3.37	1.31	4.7	25.7	18.7	38.4	6.6	2.8	3.1	
Fruit juices (100% fruit), packaged	pre	3.28	1.75	11.7	32.3	18.1	14.4	7.5	9.6	6.4	**<0.0001**
	peri	2.77	1.42	16.1	36.8	19.2	18.1	4.2	2.8	2.8	
Carbonated sugar sweetened drinks (e.g., coca cola, Fanta)	pre	1.77	1.22	56.8	27.2	7.2	3.5	2.9	1.1	1.3	**<0.0001**
	peri	1.40	0.81	71.1	22.8	3.4	1.7	0.0	0.3	0.6	
Diet carbonated drinks (e.g., diet cola)	pre	1.43	1.03	78.1	12.3	4.0	2.7	1.1	1.1	0.8	**0.0176**
	peri	1.22	0.58	83.2	13.9	1.9	0.5	0.3	0.3	0	
Sweetened or sugar added breakfast cereals	pre	2.21	1.30	34.7	34.1	16.8	10.1	0.8	1.3	2.1	0.6000
	peri	2.18	1.12	31.4	38.5	13.9	13.9	1.7	0.3	0.3	
Plain unsweetened milk	pre	2.34	1.52	35.7	30.4	17.1	8.3	2.7	1.6	4.3	**<0.0001**
	peri	2.83	1.22	14.8	28.4	22.9	29.3	2.7	0.8	1.1	
Plain unsweetened yoghurt or kefir	pre	1.71	1.11	57.1	28.5	6.9	4.8	0.3	1.6	0.8	**0.0009**
	peri	1.80	0.89	44.3	39.2	9.8	6.1	0.6	0	0	
Sweet and flavored yoghurt	pre	2.15	1.24	32.8	40.0	16.3	6.1	1.6	1.3	1.9	**0.0147**
	peri	2.23	1.03	23.9	45.4	17.5	11.5	0.9	0.5	0.3	
Fish, boiled, grilled, oven baked, raw	pre	1.52	0.90	61.6	32.3	2.4	2.1	0.5	0	1.1	**0.0058**
	peri	1.55	0.67	50.7	46.0	2.2	0.5	0.3	0	0.3	
Fish, fried and/or coated	pre	1.55	0.86	58.1	34.9	3.5	2.4	0.3	0	0.8	0.6061
	peri	1.49	0.60	54.3	43.2	1.7	0.3	0.5	0	0	
Cold cuts and preserved, ready-to-cook meat product	pre	2.94	1.51	10.9	37.9	25.1	11.2	7.2	2.4	5.3	**0.0272**
	peri	2.61	1.09	13.6	37.6	28.5	16.7	2.2	0.8	0.6	
Poultry, meat, boiled, grilled, oven baked, without coating not fried	pre	1.98	1.17	37.6	43.2	12.0	2.1	1.9	2.1	1.1	**<0.0001**
	peri	2.27	0.85	13.6	54.6	26.2	4.2	0.6	0.3	0.5	
Fried poultry, meat	pre	1.98	1.18	36.8	46.4	8.5	3.5	2.1	0.8	1.9	0.0662
	peri	1.71	0.59	35.4	58.7	5.5	0.5	0	0	0	
White bread, white roll, white crispbread	pre	3.30	1.67	10.9	27.5	25.6	10.9	13.9	4.3	6.9	**0.0092**
	peri	3.35	1.12	6.1	17.8	22.5	46.2	4.5	2.2	0.8	
Whole meal bread, dark roll, dark crispbread	pre	2.43	1.38	25.3	39.2	18.4	8.5	4.0	2.1	2.4	0.0617
	peri	2.22	1.17	31.8	35.1	16.7	13.3	1.7	1.2	0.2	
Pasta, noodles, rice and other cereals, white, refined	pre	2.18	1.22	25.6	51.5	14.1	2.9	2.7	0.5	2.7	0.0011
	peri	2.19	0.73	10.8	65.5	19.0	3.9	0.3	0.3	0.2	
Whole meal pasta, noodles, brown rice and other cereals, unrefined	pre	1.66	1.05	58.4	28.5	7.2	2.4	2.4	0.3	0.8	0.8144
	peri	1.57	0.77	55.2	35.7	7.2	1.2	0.2	0.3	0.2	
Not homemade hamburger, hot dog, kebab, wrap, falafel, sandwich	pre	1.76	1.29	58.1	26.9	6.7	2.9	1.9	1.1	2.4	**<0.0001**
	peri	1.39	0.68	69.0	25.3	4.7	0.8	0	0.2	0.2	
Nuts and seeds	pre	2.00	1.24	39.5	40.3	11.7	3.7	1.3	1.3	2.1	**0.0095**
	peri	1.76	0.90	45.2	41.3	8.0	4.5	0.3	0.3	0.3	
Snacks such as crisps, corn crisps, popcorn, etc.	pre	1.95	1.30	45.3	35.5	9.3	4.5	1.6	1.3	2.4	**0.0003**
	peri	1.56	0.65	51.2	42.9	4.8	0.8	0.3	0	0	
Snacks such as biscuits, packaged cakes, or pastries and puddings	pre	2.17	1.23	29.9	44.0	16.5	4.5	1.3	1.9	1.9	**0.0371**
	peri	2.20	0.97	21.7	50.1	17.0	9.5	1.2	0.2	0.3	

$\bar{x}$

- arithmetic mean, SD—standard deviation, *p*—*p* value, indicate significant values (*p* < 0.05). Bold values denote statistical significance at the p < 0.05 level.

## Data Availability

The data presented in this study are available on reasonable request from the corresponding author. The data are not publicly available due to restrictions e.g., their containing information that could compromise the privacy of research participants.
